# Increased Right Ventricular Cardiac Output to Left Ventricular Cardiac Output Ratio in High-Altitude Residents Without Pulmonary Hypertension: An Echocardiography Study

**DOI:** 10.33549/physiolres.935686

**Published:** 2026-04-01

**Authors:** Donghua WANG, Yunmei FANG, Xuchu WU, Jie ZHANG, Guangfei YANG, Xiaozhi ZHENG

**Affiliations:** 1 Department of Ultrasound, Minhang Hospital, Fudan University, Shanghai, China; 2 Department of Ultrasound, Yangpu Hospital, School of Medicine, Tongji University, Shanghai, China; 3 Department of Ultrasound, The Affiliated Lianyungang Hospital of Xuzhou Medical University/The First People’s Hospital of Lianyungang, Lianyungang, Jiangsu Province, China

**Keywords:** Cardiac output, Cardiac function, High-altitude, Echocardiography

## Abstract

Right ventricular cardiac output to left ventricular cardiac output (RVCO/LVCO) ratio in high-altitude residents had not been well characterized. The purpose of this study was to investigate the normal reference range for RVCO/LVCO ratio among high-altitude residents without pulmonary hypertension. Between October 2024 and June 2025, 88 consecutive high-altitude (≥4000 meters above sea level) residents without pulmonary hypertension were enrolled in this study. RVCO/LVCO ratio was quantified by transthoracic echocardiography. The difference of RVCO/LVCO ratio between high-altitude residents and low-altitude residents were compared. High-altitude residents display significantly larger right atrium, right ventricle, and pulmonary artery dimensions than low-altitude residents, with slightly thickened right ventricular free walls but slightly smaller tricuspid annular plane systolic excursion (p<0.05). RVCO, right ventricular cardiac index, and the RVCO/LVCO ratio (1.15 [0.97–1.38] vs. 0.97 [0.91–1.05], p<0.0001) was significantly greater in high-altitude residents than in low-altitude residents, but left heart size, LVCO and left ventricular cardiac index show no significant differences between groups (p>0.05). There was a negative correlation between the RVCO/LVCO ratio and altitude (r = −0.298, p < 0.0001). The regression equation was y = 8.2083 − 0.001726x, where x represents altitude and y represents the RVCO/LVCO ratio. The increased RVCO/LVCO ratio observed in high-altitude residents without pulmonary hypertension suggests that adaptive cardiac changes may be mediated by hypoxia-driven hemodynamic compensatory adjustments. These findings make RVCO/LVCO ratio a crucial indicator for detecting plateau physiological adaptation and pathological conditions in high-altitude hypoxic environments.

## Introduction

The high-altitude hypoxic environment exerts profound influences on the human cardiovascular system, among which elevated pulmonary artery pressure and right heart involvement are common changes. When lowland populations rapidly enter areas above 2,500 meters altitude, hypoxic pulmonary vasoconstriction causes increased pulmonary artery pressure, elevating right heart afterload. This physiological change is particularly significant in acute mountain sickness patients. Studies show that after acute high-altitude exposure, the right ventricular outflow tract (RVOT) and pulmonary artery diameter significantly widen, with pulmonary artery systolic pressure (PASP) rising by an average of 30 % – 50 % [1–3].

Long-term high-altitude residents can work and live normally without obvious symptoms, possibly through compensatory mechanisms such as increased hemoglobin concentration. Current clinical evaluation of cardiac function in high-altitude residents still uses lowland standards. However, high-altitude residents may undergo pulmonary vascular remodeling and cardiac functional adjustments. Under hypoxic conditions, increased pulmonary vascular resistance and elevated right heart afterload compel the body to increase right heart output to maintain oxygen delivery, forming a ‘right heart-dominant state’ [4]. The right ventricular cardiac output to left ventricular cardiac output (RVCO/LVCO) ratio maintains within a certain range. Nevertheless, current research lacks the normal reference range for RVCO/LVCO ratio, resulting in insufficient standards for early identification and intervention of right heart dysfunction in high-altitude areas.

This study aims to establish the normal reference range for RVCO/LVCO ratio in high-altitude residents without pulmonary hypertension and compare it with low-altitude residents. This will provide evidence-based standards for right heart function evaluation in high-altitude regions, serving as reference for early warning of acute mountain sickness, diagnosis of high-altitude pulmonary hypertension, and prevention/treatment of heart failure.

## Methods

### Study population

This investigation received approval from the Human Research Ethics Committee of Yangpu Hospital, School of Medicine, Tongji University (No. LL-2024-SHZRKX-001). Written informed consent was secured from every participant before enrollment, adhering to the principles of the Declaration of Helsinki (2013 revision). From October 2024 to June 2025, consecutive individuals residing at ≥4000 meters altitude with ≥3 consecutive generations of high-altitude ancestry—verified through detailed interviews confirming their parents, grandparents, and great-grandparents were all lifelong residents of the high-altitude region (≥4000 m)—who were asymptomatic and showed no detectable physical signs, were enrolled. To control for the potential confounding effects of physical activity, the low-altitude (≤5 meters altitude) subjects were therefore matched to the high-altitude group not only for age, sex, and body surface area (BSA), but also for occupational profile. Questionnaire verification confirmed that the vast majority (>85 %) in both cohorts were engaged in outdoor manual labor, primarily farming, ensuring comparable levels of daily physical exertion.

All enrolled participants underwent an extensive diagnostic assessment protocol including: (1) systematic review of cardiopulmonary history, (2) comprehensive physical examination, (3) laboratory investigations (incorporating tumor markers and myocardial enzymes), (4) electrocardiography, and (5) multimodality imaging (radiography, ultrasonography, computed tomography). Exclusion criteria were: (i) confirmed cardiopulmonary or systemic pathologies (hypertension, pulmonary hypertension, diabetes, hyperlipidemia, cardiac dysfunction, atrial fibrillation, or >mild valvular disease); (ii) smoking; (iii) current use of cardiorespiratory medications, chemotherapeutic agents, or vasoactive drugs; (iv) hepatopulmonary syndrome or pulmonary arteriovenous malformations; and (v) suboptimal echocardiographic image quality per standard guidelines.

### Data collection

Demographics, residential altitude, and partial pressure of inspired oxygen (PiO_2_) data were analyzed using retrospective participant data combined with records from the local meteorological administration and atmospheric research institute.

### Transthoracic echocardiography

**Fig. 1:**
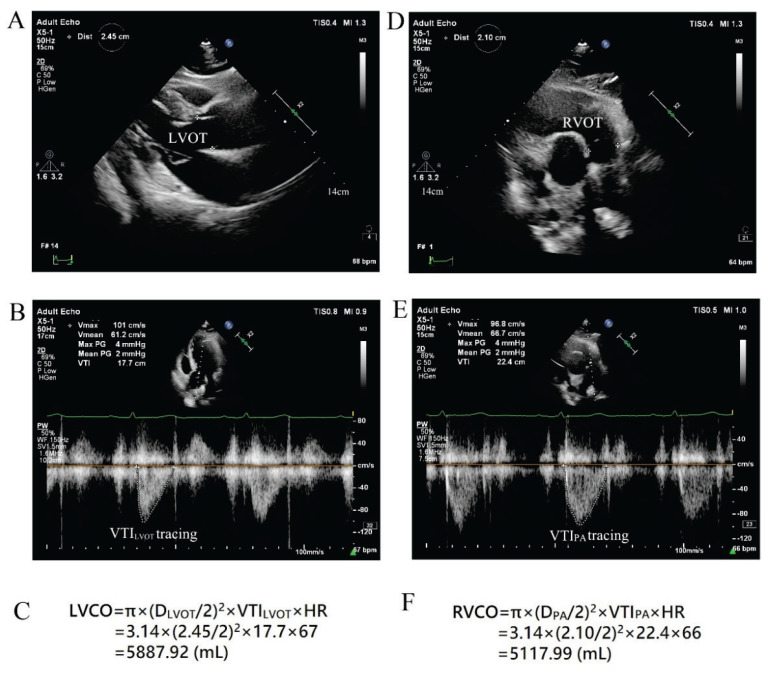
Two-dimensional and spectral Doppler images of the left ventricular outflow tract (LVOT) and pulmonary artery (PA) in a low-altitude resident, demonstrating the methodology for left ventricular cardiac output (LVCO, A-C) and right ventricular cardiac output (RVCO, D–F). D, diameter; RVOT, right ventricular outflow tract; VTI, velocity-time integral; HR, heart rate.

Transthoracic echocardiography examinations were performed using a Philips EPIQ 7C system (Netherlands) with an X5-1 PureWave xMATRIX transducer (1–5 MHz), in accordance with European Association of Cardiovascular Imaging (EACVI)/American Society of Echocardiography (ASE) recommendations [5,6]. All echocardiographic images were analyzed offline by two experienced investigators who were blinded to the participants’ group assignment (high-altitude or low-altitude). Measured parameters included: left ventricular end-diastolic anteroposterior diameter (D_LV_), right ventricular end-diastolic anteroposterior diameter (D_RV_), left atrial end-systolic transverse diameter (D_LA_), right atrial end-systolic transverse diameter (D_RA_), left ventricular ejection fraction (LVEF), left ventricular mass index (LVM index), right ventricular free wall thickness (T_RVFW_), pulmonary artery diameter (D_PA_), left ventricular outflow tract diameter (D_LVOT_), the ratio of the peak early diastolic transmitral/transtricuspid filling velocity to the peak early diastolic lateral mitral/tricuspid annulus tissue velocity (Mitral/Tricuspid E/e’), peak tricuspid regurgitation velocity (TRV_max_), left ventricular outflow tract velocity time integral (VTI_LVOT_), right ventricular outflow tract velocity time integral (VTI_RVOT_), pulmonary artery velocity time integral (VTI_PA_), and tricuspid annular plane systolic excursion (TAPSE). Using the continuity equation, stroke volume (SV) and cardiac output (CO) were calculated (Fig. 1): LVSV (mL) = π × (D_LVOT_/2)^2^ × VTI_LVOT_; LVCO (L/min) = LVSV × heart rate(HR); RVSV (mL) = π × (D_PA_/2)^2^ × VTI_PA_; RVCO (L/min) = RVSV × HR. Left and right ventricular cardiac indices (LVCI and RVCI) were calculated by dividing LVCO and RVCO, respectively, by BSA. Pulmonary artery systolic pressure (PASP) was estimated as 4×(TRV_max_)^2+^ right atrial pressure; pulmonary vascular resistance (PVR) was calculated as TRV_max_ /VTI_RVOT_ ×10 + 0.16 Wood units [7].

### Statistical analysis

The distribution of continuous variables was evaluated using the Shapiro-Wilk test. Normally distributed data were expressed as mean ± standard deviation (SD) and compared using independent samples t-tests. Data violating normality assumptions (confirmed by Shapiro-Wilk and Q-Q plots) were presented as median (interquartile range, IQR) and compared using Mann-Whitney U tests. Categorical variables were summarized as counts (percentages) and analyzed with chi-square tests. The relationship between the RVCO/LVCO ratio and altitude was evaluated through linear regression analysis. Statistical significance was defined as a two-tailed P-value < 0.05. All analyses were conducted using SPSS 19.0 (IBM Corp.) and MedCalc 16.8.4 (MedCalc Software).

## Results

### Characteristics of included patients

During the study period from October 2024 to June 2025, 32 out of 120 high-altitude residents were excluded based on pulmonary arterial hypertension criteria (PASP ≥35 mmHg), resulting in a final cohort of 88 participants (63 males and 25 females). As documented in Table 1, the high-altitude region demonstrated significantly greater altitude and lower PiO_2_ levels compared to the low-altitude region (both p<0.0001), while high-altitude residents exhibited no statistically significant differences in age, gender distribution, BSA and body mass index (p<0.05). High-altitude residents exhibit higher blood pressure and slower heart rates compared to low-altitude residents (p>0.05). Pulmonary artery pressure in high-altitude residents is slightly elevated while pulmonary resistance is significantly smaller (p<0.01), though both remain within normal ranges. High-altitude residents demonstrate significantly smaller LVEF than low-altitude residents (p<0.0001), whereas LVOT, VTI_LVOT_ and LVSV are significantly larger (p<0.05). Left heart size, LVCO and LVCI show no significant differences between groups (p>0.05).

High-altitude residents display significantly larger right atrium and right ventricle dimensions than low-altitude residents, with slightly thickened right ventricular free walls but slightly smaller TAPSE (p<0.0001). Pulmonary artery diameter, VTI_PA_, RVSV, RVCO and RVCI were all significantly larger in high-altitude residents (p<0.001). Similarly, the RVCO/LVCO ratio was significantly greater in high-altitude residents (p<0.0001).

In addition, among high-altitude residents, females exhibit significantly higher right ventricular cardiac index (RVCI) (3.22[2.60–3.74]) vs. 2.45[2.11–3.01], p<0.0001), left ventricular cardiac index(LVCI) (2.50[2.28–2.71] vs. 2.25[2.03–2.58], p=0.0065), and RVCO/LVCO ratio (1.32[1.18–1.48]) vs. 1.06[0.92–1.27], p<0.0001) compared to males.

### Relationship between RVCO/LVCO ratio and altitude in high-altitude residents

**Fig. 2:**
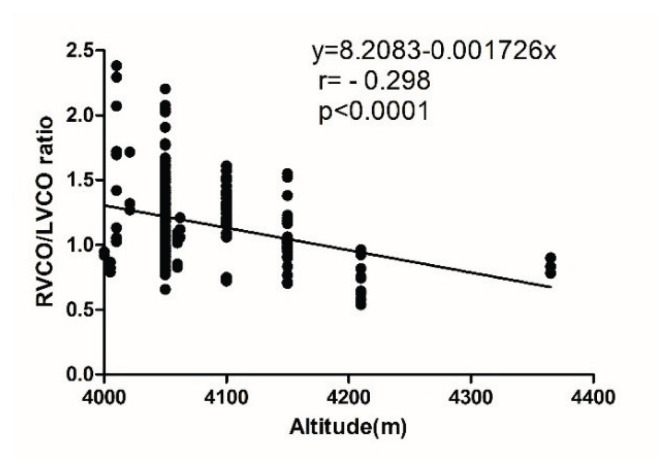
Linear regression between RVCO/LVCO ratio and altitude. LVCO, left ventricular cardiac output; RVCO, right ventricular cardiac output.

As shown in Figure 2, a negative correlation was observed between the RVCO/LVCO ratio and altitude (r = −0.298, p < 0.0001). The regression equation was y = 8.2083 – 0.001726x (R^2^ = 0.0888; 95 % confidence interval: −0.0024 to −0.0011), where x represents altitude and y represents the RVCO/LVCO ratio.

### Intra-/interobserver variability

To evaluate measurement reliability, intraobserver and interobserver variability assessments were performed. For intraobserver consistency, Observer A repeated echocardiographic parameter measurements in 30 randomly selected cases after a 3-week interval to minimize recall bias, demonstrating high reproducibility with intraclass correlation coefficients (ICC) ranging from 0.96 to 0.99 and coefficients of variation (CV) between 1.9 % and 2.3 %. Interobserver agreement was assessed through independent analyses of the same 30 cases by two observers (A and B), revealing strong concordance with ICC values of 0.94 to 0.98 and CVs of 2.1 % to 2.9 %, collectively confirming high measurement consistency across both intra- and inter-observer assessments.

## Discussion

This study is the first to establish the normal reference range for the RVCO/LVCO ratio in high-altitude residents. Establishing this range is crucial to differentiate physiological adaptation from early maladaptation, high-altitude pulmonary hypertension or high-altitude pulmonary edema. In our healthy cohort, the elevated RVCO/LVCO ratio occurs in the context of preserved RV function and the absence of pulmonary hypertension, suggesting a compensatory increase in flow. However, in the early stages of maladaptive conditions such as high-altitude pulmonary hypertension, a similarly elevated ratio might be driven by increased afterload and worsening RV function, rather than purely augmented flow. Consequently, tracking the RVCO/LVCO ratio alongside established parameters like tricuspid annular plane systolic excursion (TAPSE) and pulmonary arterial systolic pressure (PASP) could provide a more comprehensive hemodynamic profile. A rising RVCO/LVCO ratio concurrent with an increasing PASP and a declining TAPSE/PASP ratio would be highly suggestive of a transition from adaptive remodeling to pathological dysfunction [8–10]. Thus, this ratio may serve as a valuable, non-invasive tool for risk stratification and early detection of adverse outcomes in high-altitude populations.

The ‘right heart predominance’ observed in the high-altitude environment is fundamentally the result of hemodynamic compensatory adjustments. Its core mechanisms include: (i) Preload redistribution and increased right ventricular volume load: Acute high-altitude exposure triggers sympathetic nervous system activation due to hypoxia, leading to systemic venous constriction and increased venous return. Concurrently, hypoxic pulmonary vasoconstriction redistributes pulmonary blood flow, increasing perfusion (and hydrostatic pressure) in specific regions, particularly the upper lobes. This process increases right ventricular volume load. While less pronounced in multi-generational high-altitude residents compared to acutely exposed individuals, our study also suggests the presence of preload redistribution and increased right ventricular volume load, as evidenced by the enlargement of the RA, RV, RVOT, and PA observed in high-altitude residents. (ii) Differential afterload and enhanced right ventricular work: Hypoxia induces pulmonary vasoconstriction and long-term remodeling, leading to elevated pulmonary artery pressure. In this study, while the pulmonary artery systolic pressure [21.40 (13.00–30.00) mmHg] of high-altitude residents remained within the normal range, it was higher than that of low-altitude residents [19.00 (17.00–23.50) mmHg]. Left ventricular afterload remains relatively stable because systemic vascular resistance is unchanged or even decreased (high-altitude residents often exhibit peripheral vasodilation [11]). Consequently, the right ventricle must increase its contractility to maintain sufficient output, resulting in a greater increase in RVCO compared to LVCO. (iii) Reduced pulmonary vascular resistance (PVR) in healthy high-altitude residents. Our study demonstrates that PVR is significantly lower in high-altitude residents compared to low-altitude controls. This likely represents a protective factor in high-altitude adaptation, reflecting an active compensatory mechanism rather than passive tolerance. We speculate that this may represent a form of long-term, adaptive vascular remodeling. Primary reasons may include: increased synthesis of vasodilatory mediators like nitric oxide (NO) and prostacyclin (PGI_2_), downregulation of vasoconstrictive mediators like endothelin-1 (ET-1) and thromboxane A_2_ (TXA_2_), and reduced sensitivity to angiotensin II receptors [12–17]. Next, pulmonary vascular structural and functional remodeling leads to increased microvascular density and reduced basal vascular tone. Finally, enhanced ventilation-perfusion matching, increased hypoxic ventilatory response, and compensatory increases in lung volumes also contribute. Our findings show that LVEF and TAPSE were lower in high-altitude residents than in low-altitude residents, indicating that their hearts achieved a more economical cardiac compensation. Under conditions of preload redistribution, increased right ventricular volume load, and reduced pulmonary vascular resistance, relatively lower cardiac functional parameters are sufficient to eject the required stroke volume.

We also found that the RVCO/LVCO ratio was higher in females than in males. This may be attributed to greater female sensitivity to hypoxia-induced pulmonary vasoconstriction at high altitudes. Under equivalent hypoxic stimulation, the increase in right ventricular stroke volume is more pronounced in females. This finding aligns with research indicating a higher incidence of pulmonary hypertension in females [18,19]. Secondly, females exhibit higher oxygen delivery efficiency. Although female hemoglobin has slightly lower oxygen-carrying capacity, tissue oxygen utilization is more efficient, thereby reducing the necessity for excessive reliance on increased cardiac output. Thirdly, estrogen-mediated protection represents a plausible hormonal pathway, not only by directly supporting pulmonary vascular function and right ventricular contractility through the influence on key mitochondrial genes and proteins [20], but also by contributing to pulmonary circulatory optimization *via* vascular remodeling [21]. Finally, hematologic factors are also likely contributory, as females at high altitude typically exhibit a blunted erythropoietic response, resulting in lower hemoglobin concentrations and blood viscosity compared to males, which may reduce right ventricular afterload and modify cardiac output distribution.

While our high-altitude cohort as a whole exhibited a significantly elevated RVCO/LVCO ratio compared to low-altitude controls, the weak negative correlation observed across the entire study population prompts a hypothesis regarding a potential inflection point within the adaptive spectrum at high altitude. Among long-term high-altitude residents, a higher relative altitude (e.g., 4,500 m vs. 4,000 m) might be associated with a paradoxical decrease in the RVCO/LVCO ratio. This could be explained by several non-mutually exclusive mechanisms. First, with increasing altitude and worsening hypoxic stress, a point may be reached where the compensatory increase in right ventricular output is offset by a rising afterload due to subclinical pulmonary hypertension, which begins to limit stroke volume. Second, progressive erythrocytosis and increased blood viscosity at extreme altitudes could impair right ventricular filling and function, thereby curtailing the RVCO. Third, there may be a shift in the nature of pulmonary vascular remodeling from a adaptive, distensible phenotype to a more maladaptive, stiffened one, reducing pulmonary arterial compliance and ventricular-vascular coupling. Finally, the observed correlation could reflect a ‘healthy survivor’ effect, where individuals with a genetic predisposition for lower RVCO/LVCO ratios (and thus a lower right ventricular workload) are better suited to thrive at the most extreme altitudes. These speculative mechanisms warrant further investigation in larger longitudinal studies spanning a broader altitudinal gradient.

This study has several limitations. Firstly, population coverage was incomplete as it excluded extremely high-altitude residents (>4500 m)—who may exhibit higher RVCO/LVCO ratios—and underrepresented children, the elderly, and females, necessitating further investigation. Secondly, the absence of cross-ethnic comparisons among major high-altitude populations (e.g., Tibetan, Andean, Ethiopian) limits insights into gene-environment interactions. Thirdly, echocardiographic cardiac output estimation (VTI × cross-sectional area) may be less accurate than MRI quantification and depends on Doppler alignment. Finally, mechanistic and intervention studies were not conducted. Future research should explore therapies targeting the RVCO/LVCO ratio (such as selective pulmonary vasodilators or cardiac energy metabolism modulators) for treating high-altitude heart disease.

## Conclusion

Through this cross-sectional investigation, we have, for the first time, established the normal reference range for the RVCO/LVCO ratio in healthy high-altitude residents. This provides an evidence-based standard for cardiac function assessment in high-altitude regions. The study reveals that the “right heart predominance” state in the plateau environment represents an adaptive remodeling of the body to hypoxia, characterized by compensatory increases in right ventricular output, active reduction of pulmonary vascular resistance, and sex-specific regulation. These findings fill a critical gap in key parameters of high-altitude cardiovascular physiology and hold significant implications for revolutionizing high-altitude medical practice and improving the cardiovascular health of plateau residents. Future large-scale cross-ethnic cohort studies and in-depth molecular investigations are needed to further refine the theoretical framework of high-altitude cardiovascular adaptation.

## Figures and Tables

**Table 1:** Descriptive demographic, clinical, and echocardiographic characteristics of all participants

Variables	Low-altitude residents n=80)	High-altitude residents (n=88)	P-value
Altitude, m	3.90(2.00–5.10)	4060.00(4020.00–4090.00)	<0.0001
PiO_2_, kPa	21.22(21.20–21.26)	12.72(11.46–12.86)	<0.0001
Age, years	35.00(28.00–45.00)	34.50(28.00–41.50)	
Male, case (%)	56(70.00)	63(71.59)	
BSA, m^2^	1.74(1.54–1.92)	1.75(1.61–1.89)	
BMI, Kg/m^2^	22.98(20.96–25.86)	22.54(20.44–25.52)	
Heart rate, bpm	70.00(62.00–75.00)	65.00(56.00–72.00)	0.0001
SBP, mmHg	121.00(110.50–137.00)	124.00(119.50–137.00)	0.0475
DBP, mmHg	80.00(70.00–87.00)	86.00(78.00–90.00)	<0.0001
PASP, mmHg	19.00(17.00–23.50)	21.40(13.00–30.00)	0.0058
PVR, wood	1.34(1.18–1.47)	1.12(0.77–1.43)	<0.0001
D_LV_, mm	45.00(42.00–48.00)	45.00(44.00–48.00)	
D_LA_, mm	31.25(29.00–34.00)	31.00(29.00–33.00)	
LVM index, g/m^2^	75.68(69.32–80.11)	73.51(63.27–84.62)	
LVEF, %	69.50(66.00–74.00)	65.00(62.00–67.00)	<0.0001
Mitral E/e’	6.97 (5.62–9.45)	6.32(5.50–7.48)	0.094
D_LVOT_, cm	1.92(1.80–2.05)	2.00(1.87–2.11)	0.0458
VTI_LVOT_, cm	18.85(17.35–21.40)	20.0(17.45–22.55)	0.033
LVSV, mL	56.55(44.70–68.72)	60.68(52.96–70.07)	0.0103
LVCO, L/min	3.63(3.13–5.31)	4.12(3.68–4.58)	
LVCI, L/min/m^2^	2.20(1.96–2.76)	2.33(2.07–2.64)	
D_RV_, mm	20.00(18.00–22.00)	23.00(21.00–25.00)	<0.0001
D_RA_, mm	32.00(30.00–34.00)	34.00(31.00–36.00)	<0.0001
T_RVFW_, mm	3.50(3.00–4.00)	5.10(4.45–5.50)	<0.0001
TAPSE, mm	21.00(20.00–23.00)	20.00(18.50–22.00)	0.0001
Tricuspid E/e’	4.67(4.20–5.42)	4.38(3.77–5.23)	
D_PA_, cm	1.80 (1.70–2.00)	2.00(1.90–2.10)	<0.0001
VTI_PA_, cm	21.80 (19.88–23.42)	22.60 (20.10–25.10)	0.0053
RVSV, mL	58.35 (47.69–66.99)	69.27 (61.59–81.71)	<0.0001
RVCO, L/min	3.79 (3.25–4.94)	4.64 (3.90–5.53)	<0.0001
RVCI, L/min/m^2^	2.31 (1.92–2.71)	2.60 (2.22–3.63)	<0.0001
RVCO/LVCO ratio	0.97 (0.91–1.05)	1.15 (0.97–1.38)	<0.0001

Data are expressed as number (percentage) or median (interquartile range). BSA, body surface area; BMI, body mass index; bpm, beats per minute; CO, cardiac output; CI, cardiac index; D_LA_, left atrial end-systolic anteroposterior diameter; DBP, diastolic blood pressure; D_LV_, left ventricular end-diastolic anteroposterior diameter; D_LVOT_, left ventricular outflow tract diameter; D_PA_, pulmonary artery diameter；D_RA_, right atrial end-systolic transverse diameter; D_RV_, right ventricular end-diastolic anteroposterior diameter; LVCI, Left ventricular cardiac index; LVEF, left ventricular ejection fraction; LVM, left ventricular mass; Mitral/Tricuspid E/e’, the ratio of the peak early diastolic transmitral/transtricuspid filling velocity to the peak early diastolic lateral mitral/tricuspid annulus tissue velocity; PA, pulmonary artery; PASP, pulmonary artery systolic pressure; PiO_2_,partial pressure of inspired oxygen; PV, pulmonary valve; RVCI, right ventricular cardiac index; SBP, systolic blood pressure; SV, stroke volume; TAPSE, tricuspid annular plane systolic excursion; T_RVFW_, right ventricular free wall thickness.
